# Supplementary material for: Exposure to low levels of hydrogen sulphide and its impact on chronic obstructive pulmonary disease and lung function in the geothermal area of Mt. Amiata in Italy: The cross-sectional InVETTA study
Source: PLoS One. 2023 Nov 1;18(11):e0293619. doi: 10.1371/journal.pone.0293619 (PMC10619772; doi:10.1371/journal.pone.0293619)
Supplement: Attachment — Submitted filename: PONE-D-23-15844_reviewer.pdf [file pone.0293619.s002.pdf]

PLOS ONE

Exposure to low levels of hydrogen sulphide and its impact on chronic obstructive pulmonary disease and lung function in the geothermal area of Mt. Amiata in Italy: the cross-sectional InVETTA study.

--Manuscript Draft--

**Manuscript Number:**	PONE-D-23-15844
**Article Type:**	Research Article
**Full Title:**	Exposure to low levels of hydrogen sulphide and its impact on chronic obstructive pulmonary disease and lung function in the geothermal area of Mt. Amiata in Italy: the cross-sectional InVETTA study.
**Short Title:**	Impact of low levels hydrogen sulphide exposure on lung fuction in the geothermal area in Italy

Corresponding Author:

Giorgia Stoppa
University of Padova: Universita degli Studi di Padova
Padua, Padua ITALY

Keywords: Hydrogen sulphide; geothermal energy; dispersion models; cross- sectional study; COPD; lung function

Abstract:

Background

The geothermal power plants for electricity production currently active in Italy are all located in Mt. Amiata area in the Tuscany region. A cross-sectional survey was conducted in the framework of the regional project “InVETTA - Biomonitoring Survey and Epidemiological Evaluations for the Protection of Health in the Amiata Territories”, using objective measures of lung function to investigate the role of hydrogen sulphide (H2S) in affecting the respiratory health of the population living in this area.

Methods

2018 adults aged 18-70 were enrolled during 2017-2019. Home and workplace addresses of participants were geocoded. Dispersion modelling was used to evaluate the spatial variability of exposure to H2S from the geothermal power plants’ emissions. We estimated average long-term historical exposure to H2S and more recent exposure indicators. Chronic Obstructive Pulmonary Disease (COPD) was defined according to the Global Initiative for Chronic Obstructive Lung Disease (GOLD). Multivariable logistic regressions were performed to investigate associations between outcome and exposure.

Results

[illegible]e of the exposure metrics produced evidence of lung function impairments. For all [illegible]ameters analysed, except for COPD Stage I, increasing H2S exposure was negatively associated, and risk reductions were observed. In particular, a -32.8% (p=0.003) risk reduction for FEV1<80% and a -51.7% (p=0.001) risk decrease for FVC<80% were [illegible]rted.

Conclusion

Our study provides no evidence that chronic exposure to low levels of H2S is associated with decrements in pulmonary function, suggesting that ambient H2S exposure may benefit lung function.

Order of Authors:

Giorgia Stoppa

Daniela Nuvolone

Davide Petri

Letizia Centi

Powered by Editorial Manager® and ProduXion Manager® from Aries Systems Corporation

	Francesca Nisticò
	Emanuele Crocetti
	Fabio Barbone
	Fabio Voller
**Additional Information:**	
**Question**	**Response**
**Financial Disclosure**<br><br>Enter a financial disclosure statement that describes the sources of funding for the work included in this submission. Review the submission guidelines for detailed requirements. View published research articles from *PLOS ONE* for specific examples.<br><br>This statement is required for submission and **will appear in the published article** if the submission is accepted. Please make sure it is accurate.<br><br>**Unfunded studies**<br>Enter: *The author(s) received no specific funding for this work.*<br><br>**Funded studies**<br>Enter a statement with the following details:<br>• Initials of the authors who received each award<br>• Grant numbers awarded to each author<br>• The full name of each funder<br>• URL of each funder website<br>• Did the sponsors or funders play any role in the study design, data collection and analysis, decision to publish, or preparation of the manuscript?<br>• **NO** - Include this sentence at the end of your statement: *The funders had no role in study design, data collection and analysis, decision to publish, or preparation of the manuscript.*<br>• **YES** - Specify the role(s) played.<br><br>* typeset	This work was supported by regional funding provided by Tuscany Region – Direzione Generale Politiche Ambientali, Energia e Cambiamenti Climatici (Resolution N. 973 on November 10, 2014). The funding source had no role in the study design, data collection, analysis and interpretation of data, the writing of the report, and the decision to submit the article for publication
**Competing Interests**<br><br>Use the instructions below to enter a competing interest statement for this submission. On behalf of all authors, disclose any competing interests that	The authors declare that they have no known competing financial interests or personal relationships that could have appeared to influence the work reported in this paper.

could be perceived to bias this work—acknowledging all financial support and any other relevant financial or non-financial competing interests.<br><br>This statement is required for submission and **will appear in the published article** if the submission is accepted. Please make sure it is accurate and that any funding sources listed in your Funding Information later in the submission form are also declared in your Financial Disclosure statement.<br><br>View published research articles from *PLOS ONE* for specific examples.<br><br>**NO authors have competing interests**<br><br>Enter: *The authors have declared that no competing interests exist.*<br><br>**Authors with competing interests**<br><br>Enter competing interest details beginning with this statement:<br><br>*I have read the journal's policy and the authors of this manuscript have the following competing interests: [insert competing interests here]*<br><br>* typeset	
**Ethics Statement**<br><br>Enter an ethics statement for this submission. This statement is required if the study involved:<br><br>• Human participants<br>• Human specimens or tissue<br>• Vertebrate animals or cephalopods<br>• Vertebrate embryos or tissues<br>• Field research<br><br>Write "N/A" if the submission does not require an ethics statement.	The Ethical Committee of the Local Health Agency of South-East Tuscany approved the study protocol (Prot N. 10679_2017, prot N. 10679_2018). All participants gave written informed consent to the study, but failure to sign it led to exclusion. Minors were not included in the study.<br><br>Consent to Participate<br>All participants gave their informed consent to the study, failure to sign it led to exclusion from the study

Powered by Editorial Manager® and ProduXion Manager® from Aries Systems Corporation

General guidance is provided below. Consult the submission guidelines for detailed instructions. **Make sure that all information entered here is included in the Methods section of the manuscript.**<br><br>**Format for specific study types**<br><br>**Human Subject Research (involving human participants and/or tissue)**<br>• Give the name of the institutional review board or ethics committee that approved the study<br>• Include the approval number and/or a statement indicating approval of this research<br>• Indicate the form of consent obtained (written/oral) or the reason that consent was not obtained (e.g. the data were analyzed anonymously)<br><br>**Animal Research (involving vertebrate animals, embryos or tissues)**<br>• Provide the name of the Institutional Animal Care and Use Committee (IACUC) or other relevant ethics board that reviewed the study protocol, and indicate whether they approved this research or granted a formal waiver of ethical approval<br>• Include an approval number if one was obtained<br>• If the study involved *non-human primates*, add *additional details* about animal welfare and steps taken to ameliorate suffering<br>• If anesthesia, euthanasia, or any kind of animal sacrifice is part of the study, include briefly which substances and/or methods were applied<br><br>**Field Research**<br><br>Include the following details if this study involves the collection of plant, animal, or other materials from a natural setting:<br>• Field permit number<br>• Name of the institution or relevant body that granted permission	
**Data Availability**<br><br>Authors are required to make all data	No - some restrictions will apply

Powered by Editorial Manager® and ProduXion Manager® from Aries Systems Corporation

underlying the findings described fully available, without restriction, and from the time of publication. PLOS allows rare exceptions to address legal and ethical concerns. See the PLOS Data Policy and FAQ for detailed information.<br><br>A Data Availability Statement describing where the data can be found is required at submission. Your answers to this question constitute the Data Availability Statement and **will be published in the article**, if accepted.<br><br>**Important:** Stating 'data available on request from the author' is not sufficient. If your data are only available upon request, select 'No' for the first question and explain your exceptional situation in the text box.<br><br>Do the authors confirm that all data underlying the findings described in their manuscript are fully available without restriction?	
**Describe where the data may be found in full sentences. If you are copying our sample text, replace any instances of XXX with the appropriate details.**<br><br>• If the data are **held or will be held in a public repository**, include URLs, accession numbers or DOIs. If this information will only be available after acceptance, indicate this by ticking the box below. For example: *All XXX files are available from the XXX database (accession number(s) XXX, XXX.*).<br>• If the data are all contained **within the manuscript and/or Supporting Information files**, enter the following: *All relevant data are within the manuscript and its Supporting Information files.*<br>• If neither of these applies but you are able to provide **details of access elsewhere**, with or without limitations, please do so. For example:<br><br>*Data cannot be shared publicly because of [XXX]. Data are available from the*	Legal restrictions exist on sharing a de-identified data set because data contain potentially identifying and sensitive people's information (age, gender, residence, health status). Aggregated data may be requested to Daniela Nuvolone: daniela.nuvolone@ars.toscana.it

XXX Institutional Data Access / Ethics Committee (contact via XXX) for researchers who meet the criteria for access to confidential data.<br><br>*The data underlying the results presented in the study are available from (include the name of the third party and contact information or URL).*<br>• This text is appropriate if the data are owned by a third party and authors do not have permission to share the data.<br><br>* typeset	
Additional data availability information:	

Powered by Editorial Manager® and ProduXion Manager® from Aries Systems Corporation

Manuscript

Click here to access/download;Manuscript;Article_25_05_2023.docx

Exposure to low levels of hydrogen sulphide and its impact on chronic obstructive pulmonary disease and lung function in the geothermal area of Mt. Amiata in Italy: the cross-sectional InVETTA study.

Giorgia Stoppa[1¶,#a,*], Daniela Nuvolone[1¶], Davide Petri[1,#c], Letizia Centi[2], Francesca Nisticò[2], Emanuele Crocetti[1], Fabio Barbone[3], Fabio Voller[1].

[1] Unit of Epidemiology, Regional Health Agency of Tuscany, via Pietro Dazzi 1, 50141 Florence (FI), Italy

[2] Health Agency of South-East Tuscany, via Curtatone 54, 52100 Arezzo (AR), Italy

[3] Department of Medicine, Surgery and Health Sciences, University of Trieste, Strada di Fiume, 447 Trieste, Italy

[#a] Current address: Unit of Biostatistics, Epidemiology and Public Health, Department of Cardiac, Thoracic, Vascular Sciences and Public Health University of Padova, via Loredan 18, 35131 Padova (PD), Italy.

[#b] Current address: Department of Clinical and Experimental Medicine, University of Pisa, via Roma 67, 56126 Pisa (PI), Italy

¶ These authors contributed equally to this work

***Corresponding author:** Giorgia Stoppa, via Loredan 18, 35131 Padova (PD), Italy, email: giorgia.stoppa@phd.unipd.it

Abstract

Background: The geothermal power plants for electricity production currently active in Italy are all located in Mt. Amiata area in the Tuscany region. A cross-sectional survey was conducted in the framework of the regional project "InVETTA - Biomonitoring Survey and Epidemiological Evaluations for the Protection of Health in the Amiata Territories", using objective measures of lung function to investigate the role of hydrogen sulphide (H_2S) in affecting the respiratory health of the population living in this area. **Methods:** 2018 adults aged 18-70 were enrolled during 2017-2019. Home and workplace addresses of participants were geocoded. Dispersion modelling was used to evaluate the spatial variability of exposure to H_2S from the geothermal power plants' emissions. We estimated average long-term historical exposure to H_2S and more recent exposure indicators. Chronic Obstructive Pulmonary Disease (COPD) was defined according to the Global Initiative for Chronic Obstructive Lung Disease (GOLD). Multivariable logistic regressions were performed to investigate associations between outcome and exposure. **Results:** None of the exposure metrics produced evidence of lung function impairments. For all parameters analysed, except for COPD Stage I, increasing H_2S exposure was negatively associated, and risk reductions were observed. In particular, a -32.8% (p=0.003) risk reduction for FEV1<80% and a -51.7% (p=0.001) risk decrease for FVC<80% were reported. **Conclusion:** Our study provides no evidence that chronic exposure to low levels of H_2S is associated with decrements in pulmonary function, suggesting that ambient H2S exposure may benefit lung function.

Keywords

Hydrogen sulphide; geothermal energy; dispersion models; cross- sectional study; COPD; lung function

Background

The advent of industrial geothermal energy utilization in Italy dates back to the 1900s. The first geothermoelectric power plant in the world was built in 1913 in Italy, in the province of Pisa. Today, in Italy, geothermal power plants are only found in Tuscany, in the two geothermal districts of Larderello-Travale and Mt. Amiata. The over 6 billion KWh produced by the 36 geothermal electric plants satisfy more than 33% of the region's energy needs, thus giving a significant contribution to the electricity balance from renewable sources in Tuscany [1,2].

Geothermal energy is a renewable energy source consisting of a body of hot aqueous fluid and hot rock continuously produced inside the earth's crust. This energy is extracted as steam or hot water from the surface and is used to heat and cool buildings (above 20° C/Km) or generate electricity [3-6].

However, geothermal energy exploitation has some adverse environmental effects, such as potential interaction with hydrological resources, subsidence problems, potential interference with natural seismicity and landscape damage. Furthermore, the atmospheric emission of non-condensable gases remains a major environmental issue related to the use of geothermal fluids to generate electricity.

In Tuscany, the geothermal power stations release pollutants such as carbon dioxide (CO_2), methane (CH_4) and hydrogen sulphide (H_2S). In addition, lower levels of several potentially harmful substances such as nitrogen, hydrogen, ammonia, boric acid, radon, rare gases and volatile trace elements (such as mercury, arsenic and antimony) are emitted [7-11]. As the main constituent of geothermal fluids, H_2S represents a leading environmental concern for air, soil, and vegetation pollution. H_2S is a flammable, colourless water-soluble gas with a characteristic odour of rotten eggs that is detectable at concentrations as low as 7 $\mu g/m^3$. The health effects of exposure to high levels of H_2S are well known, from respiratory, eye and throat irritation (5–29 mg/m^3), to olfactory nerve paralyses (>140 mg/m^3) and coma (>700 mg/m^3) [12-14]. Conversely, the health effects associated with exposure to low levels of H_2S in communities near natural or anthropogenic sources are less clear [15,16]. The literature suggests a cardioprotective role for H_2S in cardiac arrhythmias, cardiac

fibrosis, heart failure, cardiac hypertrophy, ischaemia–reperfusion injury and myocardial infarction [17]. The WHO guidelines for protecting human health set tolerable concentration at the average value of 150 μg/m^3 over 24 h, 100 μg/m^3 over 14 days and 20 μg/m^3 over 90 days [13,14].

According to the Tuscany Region health databases, several descriptive and analytical epidemiologic studies have been conducted on the population living in the geothermal district of Mt. Amiata. Local mortality and hospitalization rates were compared with average regional rates reporting some risk excesses for respiratory diseases [18-21]. In particular, in a cohort of 33,804 residents in the Amiata area, observed for a total of 391,002 person-years, exposu[illegible]o H_2S was associated with higher mortality (years 1998-2014) and hospital discharge rates (1998-2016) for respiratory diseases, chronic obstructive pulmonary disease (COPD) and disorders of the peripheral nervous system [20].

A cross-sectional survey was conducted in the framework of the regional project "InVETTA - Biomonitoring Survey and Epidemiological Evaluations for the Protection of Health in the Amiata Territories", using objective measures of lung function to investigate further the role of H_2S in determining the observed respiratory morbidity in populations living in the Mt. Amiata area.

Materials and Methods

Ethics Statements

The Ethical Committee of the Local Health Agency of South-East Tuscany approved the study protocol (Prot N. 10679_2017, prot N. 10679_2018). All participants gave written informed consent to the study, but failure to sign it led to exclusion. Minors were not included in the study.

Study design and participants

InVETTA was a cross-sectional study enrolling participants living in the geothermal area of Mt. Amiata. Participants' recruitment started on May 19th 2017, and ended on February 19th 2019.

The sample size was calculated using data from a prior monitoring survey that was carried out towards the end of the 1990s to examine the levels of mercury and arsenic in 900 respondents' blood and urine samples. The number of patients needed to detect a difference in arsenic and mercury concentrations

was 160 and almost 1500, respectively, based on the assumption that the study would have a 90% power and a 0.05 alpha error. In order to ensure the survey's proper operation and to allow as many individuals as possible the chance to participate in the survey, the expected sample size was finally determined to be 2000 subjects in light of the extensive interest of the local public opinion on environmental hazards.

A comprehensive communication plan (traditional local media, public meetings, a website dedicated to the project, and social networks) was implemented to enhance local community participation. Selection criteria were: being 18-70 years old at the time of enrolment, possessing a phone number, and having lived in the Amiata area for at least five years. Residence was classified as follows: the "Main Area" is the one which is most affected by [illegible]ssions from geothermal plants and includes the municipalities of Abbadia San Salvatore, Piancastagnaio, Arcidosso, Santa Fiora, Castel del Piano and Castell'Azzara, whereas the "Control Area" includes Seggiano, Radicofani, Cinigiano and Castiglione d'Orcia

Initially, stratified random sampling from municipal registries was applied to obtain a representative sample of subjects by gender, age group (18-39, 40-54 and 55-70 years), municipality of residence, and exposure to geothermal emissions. Volunteers' participation was also encouraged, and the same selection criteria were applied to volunteers as for the random sampling. Trained health personnel contacted all participants, both sample and volunteers, to schedule the appointments for the spirometry and the administration of questionnaires. A face-to-face interview was administered by suitably trained staff to each participant about general information, lifestyle, medical history, and occupational exposure to improve H_2S exposure evaluation and control for confounding. The same operator conducted more than 90% of the interviews to reduce the risk of introducing interviewer-related confounding. All participants' data were coded by assigning unique identifiers.

Spirometric testing

Trained health professionals performed spirometry using the KoKo® Legend II nSpireHealth, Inc. spirometer following American Thoracic Society/European Respiratory Society performance guidelines [22]. The spirometry parameters to evaluate lung function included forced vital capacity (FVC) and forced expiratory volume in one second (FEV1). Briefly, FVC is the maximum amount of air that can be exhaled when blowing out as fast as possible at the end of the manoeuvre; FEV1 is the air volume exhaled in the first second of the manoeuvre. The ratio of the two measurements (FEV1/FVC) was calculated. Spirometric values were compared with expected values calculated with the equations recommended by the Global Lung Function Initiative, ERS Task Force [23]. The prediction equations require age, gender and height. FEV1 and FVC are expressed as percentages of predicted values. COPD was defined according to the Global Initiative for Chronic Obstructive Lung Disease – GOLD [24] and the review by Halbert et al. [25] as follows:

- COPD Stage I: FEV1/F[illegible]<70%
- COPD Stage II: FEV1/FVC<70% and the percent of the predicted value of FEV1<80%.

The following were considered as additional indicators of reduced respiratory function:

- the percent of the predicted of FEV1<80% (FEV1<80%),
- the percentage of the predicted of FVC<80% (FVC <80%).

Exposure estimation

Seven geothermoelectric power plants were activated in the Mt. Amiata area in the last decades. Table 1 summarizes the characteristics of these plants. The oldest and most polluting two, Bellavista and Piancastagnaio2 (PC2) were closed in 2000 and 2011, respectively, whereas the most recent, Bagnore 4, was constructed in 2014. Filters for abating hydrogen sulphide and mercury (AMIS technology) were progressively installed over the years.

Table 1: Characteristics of the geothermoelectric power plants in the Mt. Amiata area.

Power plant	Municipality	Power (MW)	Start	AMIS* Date of installation
Piancastagnaio 2 (PC2)	Piancastagnaio	20	1968 closed in 2011	Not installed
Bellavista	Piancastagnaio	20	1987 closed in 2000	Not installed
Piancastagnaio 3 (PC3)	Piancastagnaio	20	04/05/1990	09/06/2006
Piancastagnaio 4 (PC4)	Piancastagnaio	20	28/11/1991	23/10/2008
Piancastagnaio 5 (PC5)	Piancastagnaio	20	02/02/1996	09/06/2006
Bagnore 3	Santa Fiora	20	17/12/1998	08/02/2002
Bagnore 4	Santa Fiora	40	31/11/2014	31/11/2014

* AMIS: Filters for the abatement of H_2S and mercury

H_2S air concentrations are constantly monitored by ENEL Green Power and the Regional Agency for the Environmental Protection of Tuscany (ARPAT). From 1997 to 2014, six fixed monitoring sites were installed to record H_2S hourly concentrations.

Dispersion modelling was used to evaluate the spatial variability of exposure to H_2S, whose technical and methodological details are described in Nuvolone 2019 [20]. In brief, an integrated framework using WRF, CALMET and CALPUFF modelling was implemented. CALMET provided 3D reconstructions of wind and temperature fields starting from meteorological measurements, orography and land use data. In this application, CALMET was used to downscale the wind fields produced by WRF (Weather Research and Forecasting Model), one of the most advanced mesoscale numerical weather prediction systems. CALPUFF was applied to assess H_2S long-range transport. To evaluate average long-term historical exposure to H_2S ("past scenario"), the emissions of six geothermal plants operating in the absence of any abatement filters (AMIS) were used as input data (PC2, Bellavista, PC3, PC4, PC5 and Bagnore3 plants). The maximum average mobile concentration of H_2S calculated over 90 days (H_2S_{past}), i.e. the maximum value derived from the series of moving averages calculated over 90 days throughout the year, was selected as the most representative indicator of long-term H_2S exposure, in line with WHO guidelines.

In addition, we considered a "current emissions scenario" ($H_2S_{current}$) which used emissions from the five plants currently active in the Mt. Amiata area (PC3, PC4, PC5, Bagnore 3 and Bagnore 4), excluding the oldest ones (Bellavista and PC2).

Geographical coordinates were assigned to each home address and workplace, and all subjects were included in a geographic information system (GIS). By overlapping with orthophotos and regional technical maps, the georeferencing results were subjected to data quality analysis, verifying the degree of completeness and precision of the geocoding process.

Exposure was assigned at an individual level considering residential history, exposure levels at home and work/school locations and time spent at home and work/school. In the case of multiple residence locations, we calculated a time-weighted average exposure metric, adding for each residence location the H_2S air concentration multiplied by the time spent in each residence location and dividing by the total residence time. In addition, specific weights were introduced to account for exposure levels at home and work/school locations and time spent at work/school, distinguishing between full-time and part-time activities.

As for sensitivity analyses, we considered other exposure indicators, such as the aerial distance of home address from the nearest geothermal power plant, and the odour perception obtained from the questionnaire ("Do you habitually experience the classic 'rotten egg' smell of hydrogen sulphide?").

Data analysis

Descriptive analyses were performed and the results are reported as frequency and percentage for categorical variables and mean and standard deviation (SD) for continuous variables. In addition, Wilcoxon-type tests were performed for the continuous variables, and the Pearson chi-square test was carried out for the categorical variables.

We fitted multivariable logistic regression for dichotomic outcomes: the estimated coefficients were expressed as Prevalence Ratio (PR) and 95% confidence intervals associated with interquartile range increase in H_2S concentrations [26]. In addition, sensitivity analyses were performed, introducing categorical variables for H_2S exposure metrics, using the tertiles of the distribution. For the continuous outcomes, multivariable analyses using generalized linear models were specified using a Gamma response with a log link, to reduce the skewness of spirometric measurements and provide a

natural interpretation of regression coefficients in terms of percent change [27,28]. Known risk factors for respiratory symptoms and lung function impairment were included a priori in the multivariate models: gender, age classes (18-39, 40-54, 55-70 years), education (low, medium, high), Body Mass Index (BMI) defined as kg/height^2, physical activity (active, partial active, sedentary), pack-year (0, <15, 15-30, >30) and cardiac comorbidities. Covariates examined for entry into regression models included mode of participation in InVETTA (sample vs volunteers), residence in main and control municipalities, passive smoking exposure, alcohol consumption, home dampness/mould, wood heating, pets, occupational exposure to dust, radiation, chemicals, working in a geothermal power plant, past working in mines. Additional analyses included stratified models considering gender, age group, mode of participation, smoking habits and residence in main and control areas. Missing values were excluded from the analyses.

All the analyses were performed using the STATA software version 16 (StataCorp LP, College Station, TX, USA).

Results

In the InVETTA survey, spirometry was performed on 2018 out of 2060 participants (98%). Unfortunately, a minority of participants failed to complete the test for various reasons related to specific psychophysical conditions. Fifty-two percent of the total subjects were randomly extracted, and 978 were volunteers. Among non-volunteers, the participation rate was 41.6%. No significant differences in gender, age and average exposure to H_2S from geothermal emissions were reported between respondents and non-respondents. Conversely, volunteers and randomly selected subjects reported some differences, as shown in Table 2. Volunteers lived in the main municipalities more frequently than the random sample, had a higher education level, showed a lower percentage of current smokers, a lower frequency of overweight/obesity and were less sedentary.

Table 2: Comparison between volunteers and randomly selected subjects in the InVETTA survey.

	Total N=2018	Random Sample N=1040	Volunteers N=978	p-values*
Gender				
Men	881 (43.7)	472 (45.4)	409 (41.8)	
Women	1137 (56.3)	568 (54.6)	569 (58.2)	0.107
Age (years)				
18-39	526 (26.1)	295 (28.4)	231 (23.6)	
40-54	706 (34.9)	352 (33.8)	354 (36.2)	0.053
55-70	786 (39.0)	393 (37.8)	393 (40.2)	
Residence				
Main area	1773 (88.1)	852 (81.9)	921 (94.2)	<0.001
Control area	245 (11.9)	188 (18.1)	57 (5.8)	
Education				
Low	555 (27.5)	354 (34.1)	201 (20.6)	
Medium	993 (49.3)	506 (48.7)	487 (49.9)	<0.001
High	468 (23.2)	179 (17.2)	289 (29.5)	
Smoking				
Never smokers	904 (45.2)	467 (45.3)	437 (45.1)	
Ex-smokers	610 (30.5)	281 (27.2)	329 (34.0)	<0.001
Smokers	486 (24.3)	284 (27.5)	202 (20.9)	
BMI				
Normal	1066 (52.9)	499 (48.0)	567 (58.0)	<0.001
Overweight/obese	952 (47.1)	541 (52.0)	411 (42.0)	
Physical activity				
Active	507 (25.2)	263 (25.3)	244 (25.0)	
Partial active	732 (36.3)	330 (31.8)	402 (41.2)	<0.001
Sedentary	776 (38.5)	445 (42.9)	331 (33.8)	

* p-values from χ^2 test

Figure 1 shows a map of the study area with the home addresses of the InVETTA participants (blue dots), geothermal power plants (green stars), municipal boundaries, and output of the dispersion model related to the maximum daily moving average of H_2S calculated over 90 days, estimated according to the past emission scenario (H_2S_{past}). The highest air concentrations of H_2S occurred in the East-side of the Amiata area, in the municipalities of Piancastagnaio, Abbadia San Salvatore and Castell'Azzara.

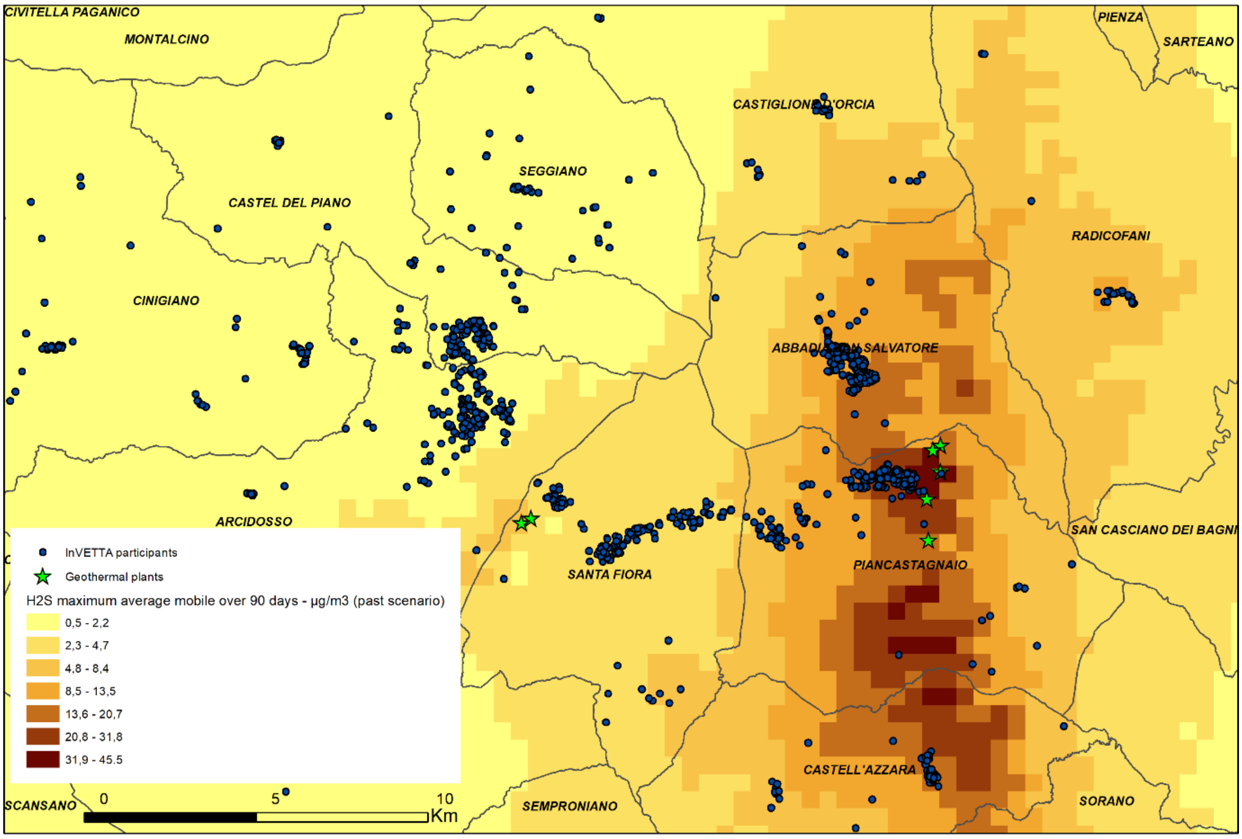

Figure 1: Map of the study area

The H_2S_{past} indicator ranged from 0.5 to 45.5 µg/m^3, with a mean of 10.4 µg/m^3 and a standard deviation of 10.5 µg/m^3. The $H_2S_{current}$ indicator showed lower values, ranging from 0.5 to 3.9 µg/m^3 with a mean of 0.8 µg/m^3 and a standard deviation of 0.7 µg/m^3. Sixteen percent of the participants were historically exposed to H_2S levels greater than the WHO guideline for medium/long-term exposure (20 µg/m^3) [13,14]. On average, InVETTA participants resided 4.5 km from the nearest geothermal power plant, ranging from 64 m to more than 23 km. About 70% of the InVETTA participants claimed to perceive the smell of H_2S. Of these, 45% reported perceiving it a few times a month, 16% every day and 10% rarely.

Descriptive results of respiratory function included a median FEV1 of 103% (SD=12.8), a median FVC of 105% (SD=12.5) and a mean FEV1/FVC of 78.0% (SD=7.5). Comparing lung function by gender and age, we found a lower breathing performance in men aged 55-70. A total of 255 subjects, i.e. 12.6% of participants, reported COPD Stage I (FEV1/FVC<70%). Of these, 63 participants (3.1%

of the total) reported COPD Stage II, and 7 subjects fell into the category ‘severe’ (FEV1/FVC<70% and 30%≤FEV1<50%). Six percent of participants, i.e. 127 subjects, reported a FEV1<80%, and 3.6%, i.e. 73 subjects, had a FVC<80%.

Table 3 reports the prevalence values of COPD and lung function indicators stratified by categories. Men showed a higher prevalence than women for COPD Stage I, whereas no difference by gender was observed for the other outcomes. Higher values were observed in the elderly, smokers, subjects with lower levels of education, subjects living in the control area, those with higher BMI, sedentary people, subjects with cardiovascular comorbidities and those who reported past working in mines.

Table 3: Prevalence of COPD and lung function outcomes (%) by categories.

	COPD Stage I			COPD Stage II			FEV1<80%			FVC<80%		
	N	%	p*	N	%	p*	N	%	p*	N	%	p*
Total	255	12.6		63	3.1		127	6.3		73	3.6	
Gender												
Men	135	15.3		28	3.2		50	5.7		29	3.3	
Women	120	10.6	<0.001	35	3.1	0.898	77	6.8	0.314	44	3.9	0.490
Age group (years)												
18-39	19	3.6		5	1.0		15	2.9		8	1.5	
40-54	69	9.8		7	1.0		29	4.1		23	3.3	
55-70	167	21.3	<0.001	51	6.5	<0.001	83	10.6	<0.001	42	5.3	<0.001
Residence												
Main area	215	12.1		47	2.7		98	5.5		58	3.3	
Control area	40	16.3	0.064	16	6.5	0.001	29	11.8	<0.001	15	6.1	0.025
Mode of participation												
Sample	132	12.7		40	3.9		72	6.9		35	3.4	
Volunteers	123	12.6	0.938	23	2.4	0.054	55	5.6	0.230	38	3.9	0.532
Pack-year												
0	79	8.7		14	1.6		38	4.2		27	3.0	
<15	68	10.4		19	2.9		38	5.8		21	3.2	
15-29	54	21.0		9	3.5		21	8.2		12	4.7	
>30	52	30.8	<0.001	20	11.8	<0.001	29	17.2	<0.001	12	7.1	0.045
Education												
Low	86	15.5		28	5.1		51	9.2		33	6.0	
Medium	117	11.8		23	2.3		53	5.3		27	2.7	
High	52	11.1	0.057	12	2.6	0.009	23	4.9	0.004	13	2.8	0.003
BMI												
Normal	129	12.1		27	2.5		55	5.2		31	2.9	
Overweight/obesity	126	13.2	0.444	36	3.8	0.107	72	7.6	0.026	42	4.4	0.071
Passive smoking												
No	227	13.0		56	3.2		108	6.2		62	3.5	
Yes	28	11.0	0.374	7	2.8	0.698	18	7.1	0.584	10	3.9	0.760
Alcohol												

Abstainer	82	12.6		24	3.7		51	7.9		29	4.5	
Moderate	144	12.9		32	2.9		59	5.3		34	3.0	
High risk	23	12.4	0.982	5	2.7	0.585	12	6.5	0.095	6	3.2	0.284
Physical activity												
Active	72	14.2		16	3.2		23	4.5		13	2.6	
Partial active	77	10.5		11	1.5		36	4.9		23	3.1	
Sedentary	105	13.5	0.097	35	4.5	0.003	67	8.6	0.002	37	4.8	0.081
Wood heating												
No	107	12.7		27	3.2		54	6.4		33	3.9	
Yes	148	12.7	1.000	36	3.1	0.884	73	6.3	0.895	40	3.4	0.567
Home dampness/mould												
No	204	13.2		47	3.0		99	6.4		57	3.7	
Yes	50	10.9	0.203	16	3.5	0.620	27	5.9	0.701	15	3.3	0.682
Pets												
No	140	14.5		28	2.9		60	6.2		44	4.6	
Yes	115	11.0	0.016	35	3.3	0.581	67	6.4	0.885	29	2.8	0.031
Occupational exposure (dust, radiation, chemicals)												
No	136	11.6		37	3.2		74	6.3		40	3.4	
Yes	119	14.1	0.097	26	3.1	0.921	53	6.3	0.973	33	3.9	0.557
Working in a geothermal power plant												
No	250	12.7		63	3.2		126	6.4		72	3.7	
Yes	5	9.8	0.537	0	0.0	0.194	1	2.0	0.197	1	2.0	0.521
Past working in mines												
No	177	11.0		47	2.9		92	5.7		52	3.2	
Yes	78	19.8	<0.001	16	4.1	0.242	35	8.9	0.020	21	5.3	0.045
Cardiac comorbidities												
No	198	11.7		45	2.7		84	5.0		47	2.8	
Sì	57	17.2	0.007	18	5.4	0.008	43	13.0	<0.001	26	7.8	<0.001

*p: p-values from χ^2 test

Descriptive statistics of H_2S indicators for both emissions scenarios stratified by outcomes are reported in Table 4. No significant differences in H_2S average concentrations were observed between subjects diagnosed with COPD, Stage I or Stage II, whereas lower H_2S levels were observed in those reporting FEV1 and FVC <80%.

Table 4: Descriptive statistics of H_2S concentrations, stratified by outcomes.

		**H_2S_{past}***		**$H_2S_{current}$****	
	N	mean (SD)	p***	mean (SD)	p***
COPD Stage I					
No	1760	10.2 (10.4)		0.80 (0.71)	
Yes	255	11.2 (11.0)	0.179	0.86 (0.75)	0.194
COPD Stage II					
No	1952	10.4 (10.42)		0.80 (0.72)	
Yes	63	9.8 (11.5)	0.139	0.73 (0.81)	0.087
FEV1%					
FEV1 >=80	1888	10.5 (10.5)	0.011	0.81 (0.72)	0.007
FEV1 <80	127	8.4 (10.2)		0.67 (0.72)	
FVC%					
FVC >=80	1942	10.51 (10.5)	0.002	0.63 (0.89)	0.002
FVC <80	73	6.7 (8.5)		0.56 (0.60)	

* H_2S_{past}: maximum average mobile concentration of H_2S calculated over 90 days according to the past emissions scenario (six geothermal power plants' emissions: Bellavista, PC2, PC3, PC4; PC5, Bagnore 3); ** $H_2S_{current}$: maximum average mobile concentration of H_2S calculated over 90 days according to the current emissions scenario (five geothermal power plants' emissions: PC3, PC4; PC5, Bagnore 3, Bagnore 4); *** p: p-values from Wilcoxon test

Table 5 shows results from generalized linear models using lung function parameters as continuous dependent variables. Percent variation and 95% confidence intervals associated with the interquartile increase of H_2S concentrations are reported, adjusting for confounding variables (gender, age, education, BMI, pack-years, physical activity, mode of participation in InVETTA and cardiac comorbidities). Past and current emissions scenarios exposure metrics were evaluated.

Table 5: Percent variation (PV) and 95% confidence interval (95%CI) in FEV1, FVC and FEV1/FVC ratio for interquartile range increases in H_2S concentration.

	H_2S_{past}*		$H_2S_{current}$**	
	PV[§]	95%CI	PV	95%CI
FEV1	1.10	0.23-1.9	0.99	0.17-1.80
FVC	1.66	0.88-2.45	1.48	0.476-2.21
FEV1/FVC	-0.51	-1.04-0.02	-0.47	-0.97-0.02

* H_2S_{past}: maximum average mobile concentration of H_2S calculated over 90 days according to the past emissions scenario (six geothermal power plants' emissions: Bellavista, PC2, PC3, PC4; PC5, Bagnore 3); ** $H_2S_{current}$: maximum average mobile concentration of H_2S calculated over 90 days according to the current emissions scenario (five geothermal power plants' emissions: PC3, PC4; PC5, Bagnore 3, Bagnore 4); [§] PV: percent variation and 95%CI from multivariate generalized linear regression models associated with interquartile range H_2S increase, adjusting for gender, age, education, BMI, pack-years, physical activity, mode of participation in InVETTA, cardiac comorbidities.

As for FEV1 and FVC parameters, slight increases were associated with increasing H_2S concentrations, +1.10% and +1.66%, respectively. On the other hand, FEV1/FVC ratio showed a very slight decrease of -0.51%, associated with increased H_2S exposure.

Table 6 shows the multivariate logistic regression results for COPD and lung function parameters using past and current emissions scenarios. Results from models using home distance from the nearest geothermal plant and odour perception are also shown. In both emissions scenarios, there was no evidence of an association between COPD and other lung function parameters and increasing H_2S exposure. For all parameters analysed, except for COPD Stage I, increasing H_2S exposure was negatively associated, and risk reductions were observed. In particular, a -32.8% (p=0.003) risk reduction for FEV1<80% and a -51.7% (p=0.001) risk decrease for FVC<80% were reported, in association with interquartile range increases in average historical H_2S concentrations. Analyses stratified by gender, age, residence, mode of participation and smoking habits confirm results observed for all participants. The highest risk reductions for FEV1<80% were observed in women, smokers and volunteers. Living near a geothermal plant or perceiving H_2S odour were not associated with increased risk for lung function. A reduced risk for COPD was reported for subjects living near a geothermal power plant. Results were also confirmed in models using the H_2S exposure metric expressed as a categorical variable (tertiles of the distribution, data not shown).

Table 6: Associations between exposures (emissions, distance from geothermal plant and perceiving H_2S odour) and outcomes (COPD and lung function parameters) from multivariate logistic regression models.

	H_2S_{past}*		$H_2S_{current}$**		Distance		Odour perception	
	PR[§]	95%CI	PR	95%CI	PR	95%CI	PR	95%CI
**COPD Stage I**								
All participants	1.039	0.900-1.199	1.032	0.900-1.183	1.058	0.965-1.160	0.830	0.654-1.055
Men	1.064	0.875-1.293	1.046	0.872-1.255	1.044	0.922-1.181	0.760	0.543-1.064
Women	1.013	0.813-1.260	1.014	0.822-1.252	1.083	0.943-1.243	0.905	0.646-1.268
18-39 years	0.873	0.442-1.721	0.763	0.394-1.476	1.416	1.050-1.911	0.747	0.308-1.808
40-54 years	1.157	0.883-1.515	1.174	0.915-1.505	0.935	0.758-1.153	0.886	0.536-1.466
55-70 years	1.028	0.866-1.220	1.016	0.846-1.197	1.072	0.963-1.194	0.837	0.633-1.106
Main area	1.114	0.954-1.301	0.110	0.958-1.287	0.907	0.748-1.101	0.894	0.680-1.177
Control area	1.429	0.503-4.060	1.391	0.546-3.542	0.951	0.769-1.176	0.763	0.441-1.321
Sample	0.994	0.841-1.176	0.986	0.839-1.157	1.087	0.979-1.208	0.762	0.560-1.038
Volunteers	1.119	0.866-1.445	1.123	0.881-1.432	0.952	0.793-1.143	0.894	0.609-1.314
Never smokers	1.166	0.901-1.510	1.122	0.886-1.420	1.087	0.909-1.300	0.638	0.415-0.979
Smokers	0.935	0.754-1.159	0.933	0.756-1.151	1.065	0.928-1.222	0.865	0.579-1.293
**COPD Stage II**								
All participants	0.783	0.565-1.086	0.789	0.570-1.093	1.231	1.055-1.436	1.030	0.616-1.720
Men	0.844	0.567-1.302	0.838	0.538-1.305	1.167	0.904-1.506	1.009	0.449-2.268
Women	0.717	0.454-1.133	0.728	0.464-1.141	1.333	1.104-1.610	1.040	0.534-2.024
18-39 years	0.192	0.026-1.407	0.166	0.019-1.450	n.d.^	n.d.	n.d.	n.d.
40-54 years	1.037	0.435-2.471	1.088	0.533-2.222	0.890	0.539-1.471	n.d.	n.d.
55-70 years	0.793	0.554-1.134	0.790	0.552-1.130	1.250	1.051-1.487	0.905	0.522-1.571
Main area	0.860	0.602-1.228	0.876	0.615-1.249	1.247	0.860-1.807	1.235	0.639-2.386
Control area	0.717	0.118-4.343	0.602	0.095-3.795	1.074	0.741-1.556	0.993	0.412-2.390
Sample	0.831	0.599-1.153	0.848	0.612-1.174	1.197	0.994-1.440	0.851	0.462-1.570
Volunteers	0.538	0.228-1.271	0.513	0.230-1.144	1.317	0.998-1.739	1.783	0.549-5.785
Never smokers	0.602	0.277-1.310	0.622	0.289-1.341	1.510	1.105-2.602	0.944	0.305-2.924
Smokers	0.735	0.452-1.194	0.723	0.441-1.185	1.266	1.006-1.594	0.969	0.401-2.342
**FEV1<80%**								
All participants	0.672	0.518-0.871	0.695	0.538-0.898	1.193	1.068-1.332	1.024	0.718-1.462
Men	0.735	0.480-1.125	0.751	0.495-1.140	1.080	0.891-1.310	1.115	0.583-2.133
Women	0.632	0.456-0.875	0.660	0.478-0.911	1.281	1.124-1.460	0.983	0.639-1.513
18-39 years	0.512	0.224-1.170	0.587	0.264-1.307	1.299	0.965-1.750	1.328	0.449-3.927

40-54 years	0.684	0.384-1.217	0.765	0.461-1.270	0.979	0.735-1.303	1.346	0.624-2.901
55-70 years	0.718	0.529-0.976	0.713	0.527-0.966	1.254	1.095-1.436	0.885	0.578-1.355
Main area	0.759	0.581-0.991	0.789	0.606-1.028	1.140	0.880-1.476	1.377	0.862-2.199
Control area	0.385	0.083-1.780	0.414	0.106-1.605	1.025	0.786-1.338	0.703	0.364-1.357
Sample	0.728	0.552-0.961	0.760	0.578-0.999	1.198	1.044-1.375	0.815	0.514-1.291
Volunteers	0.478	0.277-0.824	0.477	0.288-0.790	1.191	0.981-1.447	1.559	0.800-3.037
Never smokers	0.861	0.564-1.316	0.910	0.613-1.350	1.246	1.004-1.546	1.221	0.625-2.387
Smokers	0.605	0.394-0.930	0.602	0.384-0.943	1.261	1.072-1.482	0.667	0.363-1.225
**FVC<80%**								
All participants	0.483	0.316-0.739	0.522	0.348-0.781	1.151	0.987-1.343	1.105	0.676-1.806
Men	0.350	0.141-0.869	0.346	0.141-0.851	1.230	0.961-1.573	1.943	0.715-5.282
Women	0.543	0.335-0.881	0.607	0.386-0.955	1.104	0.901-1.352	0.868	0.481-1.568
18-39 years	0.146	0.023-0.912	0.190	0.037-0.967	1.443	0.954-2.180	1.560	0.350-6.952
40-54 years	0.705	0.389-1.278	0.759	0.0441-1.308	0.985	0.746-1.302	1.168	0.490-2.784
55-70 years	0.422	0.223-0.796	0.455	0.254-0.816	1.200	0.961-1.499	1.010	0.533-1.912
Main area	0.524	0.341-0.803	0.561	0.371-0.849	1.210	0.858-1.705	1.265	0.697-2.295
Control area	0.228	0.017-2.969	0.300	0.035-2.537	0.868	0.572-1.319	1.045	0.358-3.044
Sample	0.502	0.293-0.860	0.542	0.326-0.904	1.141	0.927-1.406	0.823	0.398-1.702
Volunteers	0.426	0.224-0.809	0.454	0.250-0.823	1.212	0.956-1.537	1.383	0.635-3.014
Never smokers	0.754	0.402-1.141	0.808	0.445-1.469	1.152	0.854-1.554	1.295	0.592-2.834
Smokers	0.294	0.123-0.705	0.303	0.132-0.696	1.306	1.017-1.677	0.505	0.185-1.382

* H_2S_{past}: maximum average mobile concentration of H_2S calculated over 90 days according to the past emissions scenario (six geothermal power plants' emissions: Bellavista, PC2, PC3, PC4; PC5, Bagnore 3); ** $H_2S_{current}$: maximum average mobile concentration of H_2S calculated over 90 days according to the current emissions scenario (five geothermal power plants' emissions: PC3, PC4; PC5, Bagnore 3, Bagnore 4); [§] PR: prevalence ratios and 95%CI from multivariate logistic regression models associated with interquartile range H_2S increase, adjusting for gender, age, education, BMI, pack-years, physical activity, mode of participation in InVETTA, cardiac comorbidities; ^n.d: not determined

Discussion

In the InVETTA cross-sectional study, exposure to low lev[illegible]of H_2S was not associated with chronic airway obstruction (COPD Stage I and Stage II) or any alteration of lung function. Results were confirmed in stratified analyses by gender, age group, smoking habits, residence area and mode of participation. Estimating spatial variability of H_2S air concentrations from dispersion modelling included both past and current emission settings. H_2S concentrations in the Amiata area have sharply declined over the years because of the installation of H_2S and mercury abatement filters and thanks to the closure of the most polluting power plants. Average H_2S concentrations estimated for each

participant in the InVETTA survey decreased from 10.4 μg/m^3 to 0.8 μg/m^3 when considering past and current emissions scenarios, both lower than WHO long-term guideline for protecting human health (20 μg/m^3) [13,14]. However, in both emissions settings, no evidence of an association with COPD was observed. In models using less complex exposure metrics, reduced lung function was not associated with living near a geothermal plant or the perception of H_2S odour.

Our findings are consistent with those reported by another study conducted in Rotorua, New Zealand, a city hosting the world's largest community that lives in an active geothermal field [29]. Authors examined spirometric parameters associated with ambient H_2S exposure and found no evidence of an association with COPD risk. In line with InVETTA results, the New Zealand study also highlighted negative associations, with evidence of better lung function in the higher exposure quartiles compared to the lowest exposure quartiles [29]. The absence of an association between respiratory outcome and exposure to H_2S is also confirmed in studies conducted in other countries [30-33]. Conversely, other studies have shown an association between H_2S exposure and a respiratory function deficit. Richardson has observed reduced FEV1/FVC levels in non-smoking sewer workers exposed to H_2S [34]. However, this study has significant limitations due to the lack of specific data regarding exposure levels to hydrogen sulphide and the confounding effect of possible exposure to other toxic substances [34]. Kilburn [35] reported significant reductions in FEV1 and FVC in a sample of 25 persons residing near an intensive swine farm, compared with 58 persons from two reference groups. In addition to the low sample size, this study has a high risk of selection bias because the exposed group was self-organizing. Furthermore, two systematic reviews concerning the effects of low-level H_2S exposure suggested no severe impact on the respiratory system and lung function [15,16].

A possible explanation of the beneficial effect of H_2S on lung function may be related to the essential role of endogenous H_2S in cellular functions and physiological and pathological processes [36]. Recently, several studies have investigated the therapeutic potential of H_2S for respiratory diseases, such as obstructive diseases, pulmonary fibrosis, emphysema, pancreatic inflammatory/respiratory lung injury, pulmonary inflammation, bronchial asthma and bronchiectasis [37-39]. Furthermore, in

the respiratory tract, endogenous H_2S has been shown to regulate important functions such as airway tone, pulmonary circulation, cell proliferation or apoptosis, fibrosis, oxidative stress and inflammation [40].

Our previous residential cohort study conducted in the Mt. Amiata area between 01/01/1998 and 31/12/2016 reported a 98% excess risk for COPD hospitalization (HR=1.98 95%CI: 1.49–2.63) in subjects exposed to an H_2S maximum 90-days moving average > 20 μg/m^3 as compared with subjects exposed to <5 μg/m^3 [20]. Although the same simulation modelling output for exposure assessment was used in the previous cohort study and the InVETTA survey, several factors may explain our different results. Firstly, the two populations are different in terms of age. In the previous cohort study, the population included all age groups, whereas, in InVETTA, the sample is 18 to 70 years old. Secondly, the InVETTA survey identified COPD cases through spirometric testing, whereas the previous cohort study was based on an administrative health database, and COPD cases were identified through hospital discharge diagnoses. For this reason, the two investigations may have included subjects at different levels of COPD severity. Indeed, in Italy, as in many other countries, managing various chronic diseases, such as COPD, has gradually shifted from a hospital-based approach to primary health care services. Thirdly, as opposed to the current InVETTA study, our previous cohort study did not include individual risk factors in multivariate models, limiting the ability to consider potential confounding.

Potential limitations of the present study, mainly in terms of selection bias, need to be considered. Among non-volunteers, the participation rate in the InVETTA survey was 46.1%, which is in line with the participation rates observed in recent epidemiological surveys. The steep decline in participation in scientific surveys is well documented, and over the last few years, many issues have been discussed to explain this declining trend [41-42]. Some central reasons may include the increase in research studies, the proliferation in marketing surveys and political polls often indistinguishable from scientific surveys, the increasing complexity of research studies demanding long follow-up, biological samples and the growing popular disillusionment with science worldwide. The potential

bias resulting from a low participation rate is a central issue in epidemiological research. Our study comparison analyses between respondents and non-respondents showed no significant differences.

In this study, we accepted volunteers to participate in the survey. This choice could have introduced a selection bias because volunteers could not represent the general population. However, comparisons between volunteers and participants randomly selected from municipal registries showed some differences: volunteers reported higher levels of education, lower percentages of smokers, they were less sedentary and overweight/obese. In addition, volunteers were less exposed to hydrogen sulphide than subjects randomly selected. For these reasons, we addressed this critical issue in the statistical protocol in various ways. First, we included in multivariate models a dichotomous variable identifying the mode of participation (sample vs volunteers) as a potential confounder, and secondly, we conducted stratified analyses to remove any confounding. Although suffering from greater imprecision due to the lower population size, stratified analyses confirm the lack of association between exposure to hydrogen sulphide and respiratory health in both subgroups.

Regarding potential information bias, in our study, the assessment of H_2S exposure was based on data from dispersion models produced according to international standards and simulation output validated using data from fixed monitoring sites. Past and current emission scenarios were included as input data in simulation models to consider both the effect of long-term exposure over the years and the effect of more recent exposure. Considering the residential history of InVETTA participants and the exposure levels in their work and study locations, including time spent at home or work, made it possible to maximize the accuracy of individual exposure estimation, minimizing the risk of introducing misclassification bias. Additional sensitivity analyses used the home distance from the nearest geothermal power plant and the odour perception as an exposure proxy. Distance is a crude approximation of exposure to ambient H_2S concentrations since it does not consider meteorological conditions, which can strongly modify the exposure levels of the resident population. Odour perception is also a very subjective indicator. The pungent odour of H_2S can cause a wide range of psychosomatic or even physiological symptoms, including respiratory complaints such as whistling

and irritation of the nose or throat [15]. However, sensitivity analyses confirm results from primary analyses, i.e. the lack of association between increasing exposure to geothermal plants' emissions and adverse respiratory health effects.

Numerous risk factors and potential confounders were evaluated and included in multivariate models. Individual information about lifestyle, habits, occupational exposure, home characteristics and clinical history were collected through a face-to-face administered questionnaire, according to the standard format used in similar cross-sectional surveys on respiratory health. Among environmental risk factors, we did not include other air pollutants typical of urban or industrial areas, such as particulate matter (PM) and nitrogen oxides (NO_2). In the Amiata area, spot surveys conducted by the Regional Agency for the Environmental Protection of Tuscany reported PM and NO_2 concentrations below the standard limit and WHO guidelines. Therefore, the potential role of these pollutants in determining the respiratory health of population is likely negligible. More relevant could be the role of arsenic and other metals, naturally present in local springs connected to the drinking water distribution system. Mt. Amiata is the second tallest volcano in Italy, and arsenic's widespread presence in groundwater is a well-known public health problem. For many years municipalities of the Mt. Amiata area requested derogations from the standard limit of As in drinking water (10 µg/l), until 2011, when huge intervention measures and structural works were put in place in order to restore water quality. An indicator of long-term exposure to arsenic in drinking water was calculated from water sampling campaigns conducted by the water utility company. Adjustment for this potential confounder did not modify our results on the relationship between respiratory outcome and H_2S exposure.

Conclusions

In conclusion, this large cross-sectional survey relies on objective and quality-controlled outcome measures, controlling for a wide set of potential confounders and comprehensive modelling of long-term and current H_2S exposures. Its findings provide no evidence that chronic and current exposure

to low levels of H_2S is associated with impairment of pulmonary function. Instead, they suggest that ambient H_2S exposure at levels measured in Mt. Amiata may benefit lung function.

Availability of data and materials:

Legal restrictions exist on sharing a de-identified data set because data contain potentially identifying and sensitive people's information (age, gender, residence, health status). Aggregated data may be requested to Daniela Nuvolone: daniela.nuvolone@ars.toscana.it

Abbreviations:

InVETTA: Biomonitoring Survey and Epidemiological Evaluations for the Protection of Health in the Amiata Territories; COPD: Chronic Obstructive Pulmonary Disease; GOLD: Global Initiative for Chronic Obstructive Lung Disease; ARPAT: Regional Agency for the Environmental Protection of Tuscany; CO_2: carbon dioxide; CH_4: methane; H_2S: hydrogen sulphide; WHO: World Health Organization; ATSDR: Agency for Toxic Substances and Disease Registry; FVC: forced vital capacity; FEV1: forced expiratory volume in one second; ERS: European Respiratory Society; WRF: Weather Research and Forecasting Model; H_2S_{past}: maximum average mobile concentration of H_2S calculated over 90 days according to the past emissions scenario; $H_2S_{current}$: maximum average mobile concentration of H_2S calculated over 90 days according to the current emissions scenario; AMIS: filters for the abatement of hydrogen sulphide and mercury: SD: standard deviation; GIS: geographical information system; PR: prevalence ratio; BMI: body mass index: PM: particulate matter; NO_2: nitrogen oxides.

References

1. Regional Agency for the Environmental Protection of Tuscany (ARPAT). Monitoraggio delle aree geotermiche toscane, anno 2019. ARPAT, Firenze; 2021.
2. Razzano, F., Cei, M. Geothermal Power Generation in Italy 2010-2014 Update Report In: Proceedings world geothermal congress 2015 Melbourne, Australia, 19–25 April 2015.
3. Barbier E, Santoprete G. L'energia geotermica. Una fonte di energia all'interno della terra. Giappichelli Editore, Torino; 1993.
4. Dickson M.H, Fanelli M..Geothermal Energy Utilization and Technology. Earthscan, London; 2003.
5. Shortall R, Davidsdottir B, Axelsson G. Geothermal energy for sustainable development: A review of sustainability impacts and assessment frameworks. Renewable and Sustainable Energy Reviews. 2015 Apr;44:391–406. https://doi.org/10.1016/j.rser.2014.12.020
6. Lund J.W, Toth A.N.Direct utilization of geothermal energy 2020 worldwide review. Geothermics. 2021; 90, 101915. https://doi.org/10.1016/j.geothermics.2020.101915
7. Regional Agency for the Environmental Protection of Tuscany (ARPAT). Monitoraggio aree geotermiche. ARPAT, Firenze; 2002.

8. Regional Agency for the Environmental Protection of Tuscany (ARPAT). Monitoraggio delle aree geotermiche: Rapporto finale biennio 2007–2008. ARPAT, Firenze; 2009.
9. Regional Agency for the Environmental Protection of Tuscany (ARPAT). Monitoraggio delle aree geotermiche toscane: Concentrazioni di idrogeno solforato nelle aree geotermiche toscane. Validazione dati ENEL e monitoraggi, ARPAT, Firenze; 2014.
10. Regional Agency for the Environmental Protection of Tuscany (ARPAT). Monitoraggio aree geotermiche. ARPAT, Firenze; 2015.
11. Somma R, Granieri D, Troise C, Terranova C, De Natale G, Pedone M. Modelling of hydrogen sulfide dispersion from the geothermal power plants of Tuscany (Italy). Science of The Total Environment. 2017 Apr 1;583:408–20. https://doi.org/10.1016/j.scitotenv.2017.01.084
12. Agency for Toxic Substances and Disease Registry (ATSDR). Toxicological profile for Hydrogen Sulphide. ATSDR, Atlanta; 2006.
13. World Health Organization (WHO). Air quality guidelines for Europe; 2 ed. WHO regional publications. 2000; 91:146–8
14. World Health Organization (WHO). Concise international chemical assessment document 53 hydrogen sulfide: human health aspects. WHO, Geneva; 2003.
15. Lewis RJ, Copley GB. Chronic low-level hydrogen sulfide exposure and potential effects on human health: A review of the epidemiological evidence. Critical Reviews in Toxicology. 2015 Feb 7;45(2):93–123.https://doi.org/10.3109/10408444.2014.971943
16. Lim E, Mbowe O, Lee ASW, Davis J. Effect of environmental exposure to hydrogen sulfide on central nervous system and respiratory function: a systematic review of human studies. International Journal of Occupational and Environmental Health. 2016 Jan 2;22(1):80–90. https://doi.org/10.1080/10773525.2016.1145881
17. LaPenna KB, Polhemus DJ, Doiron JE, Hidalgo HA, Li Z, Lefer DJ. Hydrogen Sulfide as a Potential Therapy for Heart Failure-Past, Present, and Future. Antioxidants (Basel). 2021 Mar 19;10(3):485.https://doi.org/10.3390/antiox10030485
18. Minichilli F, Nuvolone D, Bustaffa E, Cipriani F, Vigotti MA, Bianchi F. State of health of populations residing in geothermal areas of Tuscany. Epidemiol Prev. 2012; 36(5 Suppl. 1):1–104.
19. Bustaffa E, Minichilli F, Nuvolone D, Voller F, Cipriani F, Bianchi F. Mortality of populations residing in geothermal areas of Tuscany during the period 2003–2012. Ann Ist Super Sanita. 2017; 53:108–117. https://doi.org/10.4415/ANN_17_02_06
20. Nuvolone D, Petri D, Pepe P, Voller F. Health effects associated with chronic exposure to low-level hydrogen sulfide from geothermoelectric power plants. A residential cohort study in the geothermal area of Mt. Amiata in Tuscany. Science of the Total Environment. 2019;10. https://doi.org/10.1016/j.scitotenv.2018.12.363
21. Nuvolone D, Petri D, Biggeri A, Barbone F, Voller F. Health effects associated with short-term exposure to hydrogen sulfide from geothermal power plants: a case-crossover study in the geothermal areas in Tuscany. Int Arch Occup Environ Health. 2020 Aug;93(6):669–82. . https://doi.org/10.1007/s00420-020-01522-9
22. Miller M.R., Hankinson J, Brusasco V, Burgos F, Casaburi R, Coates A,et al. ATS/ERS Task Force. Standardisation of spirometry. Eur Respir J. 2005 Aug;26(2):319-38. https://doi.org/10.1183/09031936.05.00034805
23. Quanjer PH, Tammeling GJ, Cotes JE, Pedersen OF, Peslin R, Yernault JC. Lung volumes and forced ventilatory flows. Report Working Party Standardization of Lung Function Tests, European Community for Steel and Coal. Official Statement of the European Respiratory Society. Eur Respir J Suppl. 1993 Mar;16:5–40.
24. Vogelmeier CF, Criner GJ, Martinez FJ, Anzueto A, Barnes PJ, Bourbeau J, et al. Global Strategy for the Diagnosis, Management, and Prevention of Chronic Obstructive Lung Disease 2017 Report. GOLD Executive Summary. Am J Respir Crit Care Med. 2017 Mar;195(5):557–82. https://doi.org/10.1164/rccm.201701-0218PP

25. Halbert RJ, Natoli JL, Gano A, Badamgarav E, Buist AS, Mannino DM. Global burden of COPD: systematic review and meta-analysis. European Respiratory Journal. 2006 Sep 1;28(3):523–32.https://doi.org/10.1183/09031936.06.00124605
26. Zocchetti C, Consonni D, Bertazzi PA. Estimation of Prevalence Rate Ratios from Cross-Sectional Data. Int J Epidemiol. 1995;24(5):1064–5.https://doi.org/10.1093/ije/24.5.1064
27. McCullagh P, Nelder J.A. Generalized linear models, 2nd ed. ed, Monographs on statistics and applied probability. Chapman & Hall/CRC, Boca Raton.1998
28. Madsen H, Thyregod P. Introduction to General and Generalized Linear Models: CRC Press. 2010.
29. Bates MN, Crane J, Balmes JR, Garrett N. Investigation of Hydrogen Sulfide Exposure and Lung Function, Asthma and Chronic Obstructive Pulmonary Disease in a Geothermal Area of New Zealand. Carpenter DO, editor. PLoS ONE. 2015 Mar 30;10(3):e0122062.https://doi.org/10.1371/journal.pone.0122062
30. Dales R.E, Spitzer W.O, Suissa S, Schechter M.T, Tousignant P, Steinmetz N. Respiratory Health of a Population Living Downwind from Natural Gas Refineries. Am Rev Respir Dis. 1989;139, 595–600. https://doi.org/10.1164/ajrccm/139.3.595
31. Jappinen P, Vilkka V, Marttila O, Haahtela T. Exposure to hydrogen sulphide and respiratory function. Occupational and Environmental Medicine. 1990 Dec 1;47(12):824–8.https://doi.org/10.1136/oem.47.12.824
32. Mostaghni AA, Nabipour I, Dianat M, Hamidi B. Pulmonary Symptoms and Spirometric Values in Kangan Sour Gas Refinery Workers. Archives of Environmental Health: An International Journal. 2000 Sep;55(5):297–9. https://doi.org/10.1080/00039890009604020
33. Schinasi L, Horton RA, Guidry VT, Wing S, Marshall SW, Morland KB. Air Pollution, Lung Function, and Physical Symptoms in Communities Near Concentrated Swine Feeding Operations. Epidemiology. 2011 Mar;22(2):20815. https://doi.org/10.1097/EDE.0b013e3182093c8b
34. Richardson DB. Respiratory effects of chronic hydrogen sulfide exposure. Am J Ind Med. 1995 Jul;28(1):99–108.https://doi.org/10.1002/ajim.4700280109
35. Kilburn KH. Human Impairment from Living near Confined Animal (Hog) Feeding Operations. Journal of Environmental and Public Health. 2012;2012:1–11. https://doi.org/10.1155/2012/565690
36. Khattak S, Zhang QQ, Sarfraz M, Muhammad P, Ngowi EE, Khan NH, et al. The Role of Hydrogen Sulfide in Respiratory Diseases. Biomolecules. 2021 May 1;11(5):682.https://doi.org/10.3390/biom11050682
37. Chen YH, Yao WZ, Geng B, Ding YL, Lu M, Zhao MW, et al. Endogenous Hydrogen Sulfide in Patients With COPD. CHEST. 2005 Nov 1;128(5):3205–11. https://doi.org/10.1378/chest.128.5.3205
38. Chen YH, Wu R, Geng B, Qi YF, Wang PP, Yao WZ, et al. Endogenous hydrogen sulfide reduces airway inflammation and remodeling in a rat model of asthma. Cytokine. 2009 Feb;45(2):117–23.https://doi.org/10.1016/j.cyto.2008.11.009
39. Zhang J, Wang X, Chen Y, Yao W. Correlation between levels of exhaled hydrogen sulfide and airway inflammatory phenotype in patients with chronic persistent asthma. Respirology. 2014;19(8):1165–9.https://doi.org/10.1111/resp.12372
40. Bazhanov N, Ansar M, Ivanciuc T, Garofalo RP, Casola A. Hydrogen Sulfide: A Novel Player in Airway Development, Pathophysiology of Respiratory Diseases, and Antiviral Defenses. Am J Respir Cell Mol Biol. 2017 Oct;57(4):403–10.https://doi.org/10.1165/rcmb.2017-0114TR
41. Nohr E.A, Frydenberg M, Henriksen T.B, Olsen J. Does low participation in cohort studies induce bias? Epidemiology. 2006; 17, 413-8 https://doi.org/10.1097/01.ede.0000220549.14177.60
42. Galea S, Tracy M. Participation rates in epidemiologic studies. Ann Epidemiol. 2007;17, 643-53 https://doi.org/10.1016/j.annepidem.2007.03.013

Acknowledgements

We would like to thank the health professionals and administrative personnel whose valuable work was essential for the implementation of the survey.

Funding

This work was supported by regional funding provided by Tuscany Region – Direzione Generale Politiche Ambientali, Energia e Cambiamenti Climatici (Resolution N. 973 on November 10, 2014). The funding source had no role in the study design, data collection, analysis and interpretation of data, the writing of the report, and the decision to submit the article for publication

Ethical Approval

The Ethical Committee of the Local Health Agency of South-East Tuscany approved the study protocol (Prot N. 10679_2017, prot N. 10679_2018).

Consent to Participate

All participants gave their written informed consent to the study, failure to sign it led to exclusion from the study.

Declaration of competing interest

The authors declare that they have no known competing financial interests or personal relationships that could have appeared to influence the work reported in this paper.

CRediT authorship contribution statement

GS: methodology, formal analysis, interpretation and major contributor in writing the manuscript

DN: conceptualization, methodology, interpretation and major manuscipt writing

DP: data management, formal analysis, interpretation and manuscipt revision

EC: methodology, interpretation and manuscript revision

FB: methodology, interpretation and manuscript revision

FV: conceptualization, methodology, supervision and manuscript revision

All authors read and approved the final manuscript.

strobe checklist

Click here to access/download

Supporting Information

STROBE-checklist-v4-combined-PlosMedicine.docx
