# Supplementary material for: Exposure to low levels of hydrogen sulphide and its impact on chronic obstructive pulmonary disease and lung function in the geothermal area of Mt. Amiata in Italy: The cross-sectional InVETTA study
Source: PLoS One. 2023 Nov 1;18(11):e0293619. doi: 10.1371/journal.pone.0293619 (PMC10619772; doi:10.1371/journal.pone.0293619)
Supplement: Attachment — Submitted filename: Response to Reviewers.docx [file pone.0293619.s003.docx]

Journal Requirements

1. Please ensure that your manuscript meets PLOS ONE's style requirements, including those for file naming.

**We have thoroughly reviewed the PLOS ONE author guidelines and style requirements to guarantee compliance in all aspects of the manuscript.** **If you have additional recommendations regarding style requirements, please do not hesitate to share them with me.**

2. We note that you have included the phrase “data not shown” in your manuscript. Unfortunately, this does not meet our data sharing requirements. PLOS does not permit references to inaccessible data. We require that authors provide all relevant data within the paper, Supporting Information files, or in an acceptable, public repository. Please add a citation to support this phrase or upload the data that corresponds with these findings to a stable repository (such as Figshare or Dryad) and provide and URLs, DOIs, or accession numbers that may be used to access these data. Or, if the data are not a core part of the research being presented in your study, we ask that you remove the phrase that refers to these data.

**We decided to remove the entire sentence because we think it did not add significant general value to the manuscript.**

3. We note that Figure 1 in your submission contain map images which may be copyrighted. All PLOS content is published under the Creative Commons Attribution License (CC BY 4.0), which means that the manuscript, images, and Supporting Information files will be freely available online, and any third party is permitted to access, download, copy, distribute, and use these materials in any way, even commercially, with proper attribution. For these reasons, we cannot publish previously copyrighted maps or satellite images created using proprietary data, such as Google software (Google Maps, Street View, and Earth).

**We decided to remove Figure 1 because of difficulties in seeking permission from the copyright holder**

4. Your ethics statement should only appear in the Methods section of your manuscript. If your ethics statement is written in any section besides the Methods, please delete it from any other section.

**The ethics statement has been deleted from any other section and it only appears in the Methods section**

5. In your Data Availability statement, you have not specified where the minimal data set underlying the results described in your manuscript can be found. PLOS defines a study's minimal data set as the underlying data used to reach the conclusions drawn in the manuscript and any additional data required to replicate the reported study findings in their entirety. All PLOS journals require that the minimal data set be made fully available.

**Regarding the availability of the minimal dataset underlying the results described in the manuscript, I regret to inform you that it is not possible to share the minimal dataset in its entirety. This decision is based on both legal restrictions and ethical considerations.**

**The dataset used in this study contains sensitive and personally identifiable information, which is protected by legal and privacy regulations. In light of these legal and ethical constraints, I am unable to provide access to the complete minimal dataset.**

**We have added the following sentence in the data availability section:”**

**Legal restrictions exist on sharing a de-identified data set because data contain potentially identifying and sensitive people's information (age, gender, residence, health status). A de-identified data set is not possible to provide due to ethical and legal considerations.** **The authors declare that a de-identified data set from this study are available upon request directly to Dr. Daniela Nuvolone (**[**daniela.nuvolone@ars.toscana.it**](mailto:daniela.nuvolone@ars.toscana.it)**).”**

6. Please review your reference list to ensure that it is complete and correct. If you have cited papers that have been retracted, please include the rationale for doing so in the manuscript text, or remove these references and replace them with relevant current references. Any changes to the reference list should be mentioned in the rebuttal letter that accompanies your revised manuscript. If you need to cite a retracted article, indicate the article’s retracted status in the References list and also include a citation and full reference for the retraction notice.

**We reviewed the reference list. It’s complete and correct**

**Reviewers' comments**

1. “None of the exposure metrics produced evidence of lung function impairments. For all parameters analysed, except for COPD Stage I, increasing H2S exposure was negatively associated, and risk reductions were observed”: 'negatively associated' with what? “In particular, a -32.8% (p=0.003) risk reduction for FEV1<80% and a -51.7% (p=0.001) risk decrease for FVC<80% were reported”: Exposure more that what is associated with risk reduction?

**We rewrite the sentence as follows in the Astract:**

**Our findings did not showed any evidence of an association between increasing H2S exposure and lung function impairments. Some risk reductions were observed: a -32.8% (p=0.003) for FEV1<80% and a -51.7% (p=0.001) risk decrease for FVC<80% were associated with interquartile increase (13.8 µg/m3) of H2S levels.**

2. What level of exposure: In particular, in a cohort of 33,804 residents in the Amiata area, observed for a total of 391,002 person-years, exposure to H_2_S (H2S indicator ranged from 0.5 to 33.5 μg/m) was associated with higher mortality (years 1998-2014) and hospital discharge rates (1998-2016) for respiratory diseases, chronic obstructive pulmonary disease (COPD) and disorders of the peripheral nervous system [20].

**We added the range of H2S between parenthesis: exposure to H_2_S (H2S indicator ranged from 0.5 to 33.5 μg/m)**

3. Residence was classified as follows: the “Main Area” is the one which is most affected by emissions from geothermal plants and includes the municipalities of Abbadia San Salvatore, Piancastagnaio, Arcidosso, Santa Fiora, Castel del Piano and Castell’Azzara, whereas the “Control Area” includes Seggiano, Radicofani, Cinigiano and Castiglione d’Orcia. 'Most affected' is a vague term. What is quantified as 'most'?

**We have added the following sentence in study design:” Regarding geothermal plants emissions, the distinction between “Main area” and “Control area” was based on the air H_2_S concentrations estimated using dispersion models output. The validity of dispersion models was tested against data from air quality monitoring stations present in the area (Nuvolone et al.** [**2019**](https://link.springer.com/article/10.1007/s11356-022-24953-y#ref-CR53)**).”**

4. The prediction equations require age, gender and height. FEV1 and FVC are expressed as percentages of predicted values. COPD was defined according to the Global Initiative for Chronic Obstructive Lung Disease – GOLD [24] and the review by Halbert et al. [25] as follows:

- COPD Stage I: FEV1/FVC<70%. Is it post bronchodilator?

**Considering this initial exploratory screening study, a decision has been made to refrain from administering the bronchodilator.**

**We added the following sentence in the discussion section:**

**“Another possible limitation was not performing spirometry testing post-bronchodilator; the choice was based on the nature of the study as it was a first-level screening survey.”**

5. In the InVETTA cross-sectional study, exposure to low levels of H2S was not associated with chronic airway obstruction (COPD Stage I and Stage II) or any alteration of lung function

What is the definition of 'Low level'?

**We added the following sentence in the discussion section:**

**“In the InVETTA cross-sectional study, exposure to low levels (WHO guideline [13,14]) of H_2_S, in the range 0-45 µg/m^3^ , was not associated with chronic airway obstruction (COPD Stage I and Stage II) or any alteration of lung function. Results were confirmed in stratified analyses by gender, age group, smoking habits, residence area and mode of participation. Estimating spatial variability of H_2_S air concentrations from dispersion modelling included both past and current emission settings. H_2_S concentrations in the Amiata area have sharply declined over the years because of the installation of H_2_S and mercury abatement filters and thanks to the closure of the most polluting power plants. Average H_2_S concentrations estimated for each participant in the InVETTA survey decreased from 10.4 μg/m^3^ to 0.8 μg/m^3^ when considering past and current emissions scenarios, both lower than** **WHO long-term guideline for protecting human health (20 μg/m^3^); only 16% of the study population has been previously exposed to levels exceeding 20 μg/m^3^ [13,14].”**
